# Impact of sitting at work on musculoskeletal complaints of German workers - results from the study on mental health at work (S-MGA)

**DOI:** 10.1186/s12995-024-00408-7

**Published:** 2024-03-27

**Authors:** T. H. An Dang, Karla Romero Starke, Falk Liebers, Hermann Burr, Andreas Seidler, Janice Hegewald

**Affiliations:** 1 Institute and Policlinic of Occupational and Social Medicine (IPAS), Faculty of Medicine, Technische Universität Dresden, Dresden, Germany; 2 Division 3: Work and Health, Federal Institute for Occupational Safety and Health (BAuA), Berlin, Germany

**Keywords:** Occupational sitting, Longitudinal studies, Musculoskeletal, Pain, Physical work, Sedentary

## Abstract

**Introduction:**

Sedentary behavior (including prolonged sitting) is a form of physical inactivity that has a negative impact on health, possibly including musculoskeletal complaints (MSCs). The purpose of this study was to determine the extent to which time spent sitting at work is associated with the one-year prevalence of MSCs in the neck, shoulder, upper back/thoracic spine, and lower back among workers from the Study of Mental Health in the Workplace (S-MGA). In addition, the study also examined whether leisure time, physical activity, and sex modify the relationship between occupational sitting and MSCs.

**Methods:**

For this analysis, we used the S-MGA, a 5-year prospective study in Germany. The S-MGA is a nationwide representative employee cohort study with a baseline survey in 2012 and a follow-up survey in 2017. Sitting at work was measured using a question asked at baseline. The Nordic Musculoskeletal Questionnaire was used to determine the one-year prevalence of MSCs in the neck, shoulder, upper back, and lower back pain (yes/no). The assessment of MSCs was only conducted at the 2017 follow-up. Adjusted Poisson regression models were used to determine the association of baseline level of weekly hours spent sitting at work with MSCs during follow-up. In addition to unadjusted models, models were adjusted for demographic (age, sex, body mass index and occupational skill level), occupational (heavy lifting at work), psychological disorders and lifestyle factors (smoking status and leisure time physical activity), as well as preexisting musculoskeletal conditions reported at baseline. To examine whether the relationship between sitting time and pain was modified by sex and leisure time physical activity, the models were stratified for both these variables.

**Results:**

Among the participants analyzed (*n* = 2,082), 49.8% were male, while 50.2% were female, and more than 60% of the study population spent over half of their working hours in a sitting position. Exposure to increased sitting at work reported at baseline was not consistently associated with 12-month prevalence of MSCs in the upper body at follow-up. However, differences in the association between occupational sitting and MSCs were dependent on the intensity of leisure time physical activity. Prevalence ratios (PRs) indicated an increased prevalence of MSC in the neck (PR = 1.46; 95% CI = 1.18–1.80) and shoulder (PR = 1.30; 95% CI = 1.03–1.64) in workers without leisure time physical activity who spent 25 to < 35 weekly working hours sitting.

**Discussion:**

These findings suggest that leisure time physical activity interacts with the relationship between sitting at work and MSCs. The relationship between sitting at work and musculoskeletal pain needs further investigation, but we found indications that leisure time physical activity may counter the effects of sitting at work.

**Supplementary Information:**

The online version contains supplementary material available at 10.1186/s12995-024-00408-7.

## Introduction

In industrially developed countries, musculoskeletal disorders are a leading cause of disability and incur high costs [1–3]. Work-related musculoskeletal disorders are defined as damage or diseases of muscles, nerves, tendons, joints, and cartilage or intervertebral discs due to risk factors in the work environment [4]. Work-related musculoskeletal disorders and complaints, such as neck and low back pain, remain frequent and are also a common cause of work disability [2, 5–7]. Musculoskeletal complaints (MSCs) have also been identified by the International Labor Organization (ILO) and the World Health Organization (WHO) as an occupational epidemic [8]. While heavy physical workload is known to increase the risk of developing MSCs [9, 10], prolonged sitting has also been suggested as a potential risk factor for work-related musculoskeletal disorders [11].

Sitting occupies a significant portion of our daily waking time. Globally, adults spend an average of 6.4 h per day sitting, and studies with objective measurements have shown that 50% of the European population sits more than 6 h per day [10–12]. Epidemiologic studies suggest that higher levels of sedentary work could be associated with several musculoskeletal conditions, including back and neck/shoulder pain [12]. For example, Celik et al. [13] found that among office workers, prolonged sitting at a desk, poor ergonomic working conditions/body position, work stress and lack of regular exercise were associated with lower back, upper back, shoulder, neck, arm or foot pain. However, studies examining the association between sitting time and neck and shoulder symptoms in office workers are inconsistent [12, 14–16]. Some cross-sectional studies have documented a positive association between occupational sitting and neck/shoulder pain and low back pain [17, 18]. However, longitudinal studies are sparse and show conflicting results [15, 19]. Thus, systematic reviews of longitudinal cohort and case-control studies find no association between sedentary time and the prevalence of musculoskeletal disorders [20–25]. Regarding musculoskeletal pain, systematic reviews report either positive [20, 26] or negative [27] associations between sitting time and musculoskeletal pain intensity. On the other hand, a dose-response relationship between the number of hours worked at a computer workstation and the risk of MSCs, particularly shoulder/neck, back, and upper limb pain and symptoms, has been reported by systematic reviews [28, 29].

The conflicting results may in part reflect inherent challenges in studying the association between sitting at work and MSCs. That is, well-established work-related risk-factors for MSCs, such as awkward body postures and heavy lifting at work [30] are likely to be negatively associated with sedentary work. Also, Sattar and Preiss [31] report that causality between sedentary behavior and MSCs is difficult to establish because pain and chronic disease may predispose to excessive sedentary behavior. The mixed results described above may also be due to a compensating effect of leisure time physical activities. There are studies indicating that workers with higher occupational sitting time sit less in their leisure time, i.e., workers with mostly sitting jobs were significantly more likely to be active during their leisure time than workers with more active jobs [32, 33]. Further complicating things, engaging in both a low and a high level of physical activity was associated with an increase of low back pain in a cross-sectional analysis [34]. Therefore, it seems important to consider leisure time physical activity when assessing the association between occupational sitting and MSCs.

The aim of the present study was to investigate the association between sedentary time at work and the one-year prevalence of MSCs using from the first two waves of the Study of Mental Health at Work (S-MGA). The S-MGA is prospective study of workers from across Germany designed to examine the connection between working conditions, mental health and functional ability in 5-year intervals [35]. We hypothesize that the increasing duration of sedentary behavior at work is associated with the prevalence of MSCs at follow-up, and that this relationship is modified by sex as well as leisure time physical activity.

## Materials and methods

### Study population

The data used in this study is from S-MGA, a nationally representative employee cohort study with a baseline survey in 2012 and a follow-up survey in 2017. The baseline measurements conducted in 2012 included questions about work experiences, health-related behaviors, and health status, and the follow-up survey in 2017 used the same internationally established instruments as the baseline survey [35].

The S-MGA sampling was based on the German Integrated Employment Biographies (IEB), which combines data from various sources of the German Federal Employment Agency [36]. The IEB captures employees subject to social security contributions in Germany, which accounted for more than 80% of all employees in Germany on the sampling date (December 31, 2010) [37]. The S-MGA used a two-stage cluster sampling procedure, with a random selection of 206 municipalities in the first stage and a random selection of individuals within municipalities in the second stage. At the outset (year 2012), the target population was defined as all employees subject to social security contributions in Germany born between 1951 and 1980. Civil servants, the self-employed and freelancers are not subject to social security contributions and were not included in the study [35].

**Fig. 1:**
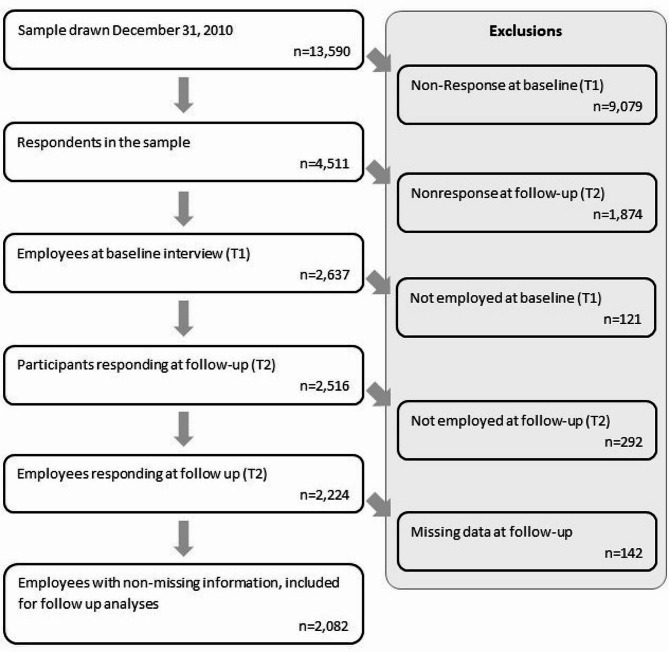
Flow of participation in S-MGA

A total of 13,590 individuals were randomly selected and then sent a letter with information on the study about a week prior to the first attempt at an in-person interview [35]. Of the 4,511 respondents who took part at baseline (response: 33%), 2,637 also took part in the follow-up interviews (follow-up: 58%) (Fig. 1). Of these, 2,224 were employed at baseline and follow-up. The study was conducted in accordance with the Declaration of Helsinki, and the protocol was approved by the ethics committee of the German Federal Institute for Occupational Safety and Health [38]. All information was obtained through computer-assisted personal interviews at the respondents’ home and all participants gave informed consent to participate in the study prior to enrollment [35].

For this analysis, we conducted a complete case analysis and excluded 142 participants with missing information on sex, age, occupational skill level, smoking, musculoskeletal diseases, leisure time physical activity, psychological conditions, working hours per week, sitting, and lifting at the workplace, and body mass index (BMI), or MSCs symptoms (neck, shoulder, upper back, and lower back) at follow-up. Included in the number of excluded participants with missing information were two persons reporting more than 90 working hours per week and 86 persons reporting highly fluctuating or irregular working hours. Application of these exclusion criteria resulted in a final sample of 2,082 participants.

The data of the first two waves of the S-MGA are available as a Scientific Use File (SUF) for scientific research projects. Access was provided by the Research Data Center of the Federal Institute for Occupational Safety and Health (FDZ-BAuA) [39].

### Variables

#### Outcome

To determine which body regions were affected by musculoskeletal symptoms in the 12-months preceding the survey, an adaptation of the Nordic Musculoskeletal Questionnaire was used [40]. The questions cover nine body regions (neck, shoulder, elbow, wrists/hands, upper back/thoracic spine, lower back (low back), hips/thighs, knees, ankles/feet) and ask: “Have you had any discomfort or pain in the following areas of your body at any time during the past 12 months?” with two response categories “yes/no”. These questions were only asked at the follow-up in 2017. The current study focused on the four upper-body regions as the dependent variables, namely, the neck, shoulders, upper back, and lower back. Studies show that upper extremities are often associated with MSC in office workers [29, 41]. The outcome in this study was the presence of MSCs in any of these four body regions during the last 12 months prior to the follow-up [40].

#### Independent variables

All independent variables used were collected at the baseline survey.

##### Occupational sitting

In the questionnaire, sitting at work was measured with a scale adapted from the BiBB/BAuA employment study [42]: “How often do you have to sit at work?” with answer choices of “never”, “up to 1/4 of the time”, “up to half of the time”, “up to 3/4 of the time”, “more than three quarters (almost all of the time)”. The assessment was performed at both baseline and at the 5-year follow-up. Only the baseline occupational sitting was considered. This information was combined with weekly working hours to obtain categories of the hours spent sitting at work per week. Weekly working time was collected with the following two questions: “How many hours a week do you normally work, including regular overtime, overtime, etc.?” and for people with a second job(s) “How many hours do you usually work there each week?”. All participants reported the sum of time (hours, minutes) they spent sitting at work on a usual working week. The hours worked were multiplied by the proportion of time spent sitting at work. Proportions equidistant from the upper and lower limits of each category, using 100% as the upper limit of the highest category were used (e.g., 0 for never and 12.5% for the category “up to 1/4 of the time”). Finally, the weekly sitting time at work were categorized as, “under 5 hours/week “, “5 to < 15 hours/week “, “15 to < 25 hours/week”, “25 to < 35 hours/week” “35 or more hours/week”.

#### Covariates

Individual factors were chosen *a priori* as potential confounders based on previous literature and theoretical assumptions concerning their possible influence on sitting behaviors and MSCs. Numerous studies have shown that working long hours increases the risk of pain or discomfort in the back, knees, hips, feet, hands, arms, neck and shoulders [43]. Sitting time at work also increases with working hours [18]. Therefore, to account for the fact that occupational sitting time is increased with increased working time, “working hours per week” was integrated into the exposure variable.

There are studies indicating a relationship between workplace sitting and age, sex [44], occupational skill level [45], working hours [46], BMI [47], smoking [18], musculoskeletal diseases [48], psychological disorders and leisure time physical activity [20, 49]. There are also studies indicating a causal relationship between MSCs and the above-mentioned factors [50–54], making them likely confounders. The DAGitty tool was used to evaluate potential confounders using directed acyclic graphs (DAG) [55]. Based on the DAGs (online supplement), we adjusted for the baseline values of age, sex, occupational level, BMI, smoking, musculoskeletal diseases, psychological disorders, lifting/carrying heavy loads at work and leisure time physical activity.

Lifting and carrying at work was assessed with a question based on a question used in the BiBB/BAuA employment study: “Lifting and/or carrying heavy loads (women more than 10 kg/ men more than 20 kg), with the answer categories “never”, “up to 1/4 of the time”, “up to half of the time”, “up to 3/4 of the time”, “more than three quarters (almost all of the time)” [42]. We adjusted for lifting and carrying because physical work done in combination with periods of sitting may be more strongly associated with MSCs. Thus, we used the variable for lifting/carrying as a proxy for more physical work.

The presence of musculoskeletal diseases at baseline was assessed using a question from the Work Ability Index (WAI): “Has a doctor ever diagnosed you with any of the diseases or health conditions listed here?” with two response categories (‘yes’ or ‘no’) [56]. A list of medical conditions including “musculoskeletal diseases of the back, limbs or other parts of the body (e.g. repeated pain in joints or muscles, sciatica, rheumatism, spinal disorders)” was shown during the personal interviews.

For sex, participants had the option of assigning themselves to “female” or “male”. Age (years) at baseline was categorized by birth year as follows: 1951-55, 1956-60, 1961-65, 1966-70, 1971-75, 1976-80. BMI was calculated using self-reported weight (kg) and height (cm) and categorized into underweight (< 18.5 kg/m^2^), normal weight (18.5–24.9 kg/m^2^), overweight (25.0–29.9 kg/m^2^), and obesity (≥ 30.0 kg/m^2^) [57, 58].

Smoking status was asked with the response options “never”, “used to smoke”, “stopped in the last twelve months”, “occasionally”, and “daily”.

Occupational skill level of work was operationalized via the respondents’ occupations, which were coded manually according to the International Standard Classification of Occupations (ISCO 08) and categorized into four groups based on skill levels: unskilled workers, skilled workers, semi-professionals, academics/managers [59].

Psychological disorders (i.e., depression, anxiety, chronic insomnia, burnout) were assessed with the above mentioned list of the WAI questionnaire used to assess musculoskeletal diseases [56]. This list also included an item for “Psychological disorders (e.g. depression, anxiety, chronic insomnia, burnout syndrome)”.

Based on the 2020 WHO guidelines on physical activity and sedentary behavior, all adults should undertake regular physical activity. Adults should do at least 150–300 min of moderate-intensity aerobic physical activity, or at least 75–150 min of vigorous-intensity aerobic physical activity, or an equivalent combination of moderate-intensity and vigorous-intensity activity throughout the week for substantial health benefits. Adults should also do muscle-strengthening activities at moderate or greater intensity that involve all major muscle groups on two or more days a week, as these provide additional health benefits [60]. In this study, we examined a single item on leisure time physical activity: “How often do you practice in activities involving more physical exertion: e.g., heavy gardening in your free time?”. The response options were “(almost) daily”, “several times a week”, “once a week”, “less often”, “never”. These variables were measured via self-reported questionnaires at baseline. We combined responses for physical activity to obtain three categories:

- mostly/all of the time = “(almost) daily” and “several times a week”,
- some/little of the time = “once a week” and “less often”, and
- none of the time = “never”.

#### Statistical analyses

Descriptive statistical procedures were performed, and inferential analysis of associations were conducted with regression analyses. Descriptive data are presented as mean and standard deviation (SD), or as frequency and percentage, where appropriate. The methods and measurement tools used are described in detail below.

Robust Poisson regression models were calculated to answer the research questions with prevalence ratios (PR) and 95% confidence intervals (95% CI). In the regression models, weekly sitting hours at work was used as the primary independent variable, with “under 5 hours/week” as the reference category. The presence of each MSC in one of the four regions of the MSCs at follow-up were the dependent variables (outcomes) and were treated separately for each outcome as binomial variables (yes/no) in all models.

Three adjustment sets were used:

- Model 1: unadjusted model.
- Model 2: an adjustment set of Model 1 with independent variables age, sex, and occupational skill level.
- Model 3: an adjustment set of Model 2 with BMI, smoking, musculoskeletal diseases at baseline, psychological disorders, lifting/carrying heavy loads at work and leisure time physical activity.

Three different models were conducted in this study; however, the main focus is the fully adjusted model (Model 3), which will be comprehensively presented in the results and discussion.

To test whether sex and leisure time physical activity modifies the effect of occupational sitting on MSCs, two two-way interaction terms (occupational sitting × sex and occupational sitting × leisure time physical activity) were included in the adjusted models. The difference in the model-fit of models with or without the two-way interaction term were tested to determine if the interaction made a statistically significant improvement to the model-fit. This would indicate effect modification. Additionally, to determine whether sex and leisure time physical activity modify the effects of prolonged sitting on MSCs risk, stratification of these models for sex (women or men) and leisure time physical activity (mostly/always, little/sometime, or never) were also performed.

The significance level was set at α = 0.05 and statistical analysis was performed syntax-based using Stata 14 [61].

## Results

Among the participants, 1036 individuals (49.8%) were male, while 1046 (50.2%) were female. The proportion of age and musculoskeletal system disease was similar between men and women. The mean working hours per week was recorded as 43.1 (SD 9.0) hours in men and 31.8 (SD 13.2) hours per week in women. Sex-related variations were evident when examining the percentage of participants meeting the WHO definition for being overweight, with 55.0% of men exceeding this threshold compared to 30.7% of women. Similarly, the prevalence of obesity among participants also exhibited sex disparities, with 19.7% of men surpassing the WHO-defined threshold as opposed to 15.6% of women.

Among both men and women participants, skilled workers accounted for 44.4% of men and 35.9% of women. Regarding psychological disorders (e.g., depression, anxiety, chronic insomnia, burnout), the minority of participants (9.2% of men and 18.0% of women) reported diagnoses at baseline. A total of 61.9% of men and 50.2% of women reported infrequent leisure time physical activity with greater physical exertion, some while approximately 9.2% of men and 5.7% of women reported leisure time physical activity with greater physical exertion more than once a week.

In terms of workplace-related factors, a considerable proportion of the sample (83.2%) reported being exposed to sitting during work. Male employees were more likely to have load lifting during working time than female employees.

In general, the proportion of MSCs (neck, shoulders, and upper back) in women was greater than that in men (64.4%, 57.7% and 35.8%, respectively). The proportion of complaint of lower back was similar between men and women (56.1% and 57.0%). These and further population characteristics are shown in Table 1.

**Table 1:** Characteristics of the sample of male and female employees

Characteristics	No. participants n (%)	Male n (%)	Female n (%)
**Total**	*n* = 2082	*n* = 1036	*n* = 1046
**Age (years)**			
31–35	193 (9.3)	108 (10.4)	85 (8.1)
36–40	310 (14.9)	153 (14.8)	157 (15.0)
41–45	449 (21.6)	221 (21.3)	228 (21.8)
46–50	491 (23.6)	240 (23.2)	251 (24.0)
51–55	421 (20.2)	206 (19.9)	215 (20.6)
56–60	218 (10.4)	108 (10.4)	110 (10.5)
**BMI**			
Underweight - BMI under 18.5 kg/m²	25 (1.2)	2 (0.2)	23 (2.2)
Normal weight - BMI greater than or equal to 18.5 to 24.9 kg/m²	799 (38.4)	260 (25.1)	539 (51.5)
Overweight - BMI greater than or equal to 25 to 29.9 kg/m²	891 (42.8)	570 (55.0)	321 (30.7)
Obesity - BMI greater than or equal to 30 kg/m²	367 (17.6)	204 (19.7)	163 (15.6)
**Occupational level**			
unskilled workers	121 (5.8)	39 (3.8)	82 (7.8)
skilled workers	835 (40.1)	460 (44.4)	375 (35.9)
semi-professionals	580 (27.9)	235 (22.7)	345 (32.9)
academics/managers	546 (26.2)	302 (29.1)	244 (23.3)
**Psychological conditions**			
Yes	283 (13.6)	95 (9.2)	188 (18.0)
No	1799 (86.4)	941 (90.8)	858 (82.0)
**Leisure time physical activity**			
mostly/all the time	155 (7.4)	95 (9.2)	60 (5.7)
some/little of the time	1166 (56.0)	641 (61.9)	525 (50.2)
None of the time	761 (36.6)	300 (29.0)	441 (44.1)
**Smoking**			
Never	852 (40.9)	390 (37.6)	462 (44.2)
In the past	622 (29.9)	337 (32.5)	285 (27.3)
Stopped in the last 12 months	37 (1.8)	24 (2.3)	13 (1.2)
Sometimes	134 (6.4)	66 (6.4)	68 (6.5)
Every day	437 (20.99)	219 (21.1)	218 (20.8)
**Musculoskeletal disease**			
Yes	1,141 (54.8)	572 (55.2)	569 (54.4)
No	941 (45.2)	464 (44.8)	477 (45.6)
**Sitting at work**			
Never	350 (16.8)	182 (17.6)	168 (16.1)
up to ¼ of the time	422 (20.3)	204 (19.7)	218 (20.8)
up to ½ of the time	344 (16.5)	177 (17.1)	167 (16.0)
up to ¾ of the time	346 (16.6)	173 (29.0)	173 (16.5)
more than ¾ the time, almost always	620 (29.8)	300 (29.0)	320 (30.6)
**Sitting at work (hours/week)**			
Under 5 h/week	570 (27.4)	243 (23.5)	327 (31.3)
5 to < 15 h/week	557 (20.4)	194 (18.7)	231 (22.1)
15 to < 25 h/week	365 (17.5)	161 (15.5)	204 (19.5)
25 to < 35 h/week	334 (16.0)	171 (16.5)	163 (15.6)
35 or more hours/week	388 (18.6)	267 (25.8)	121 (11.6)
**Heavy lifting at workplace**			
never	1,165 (56.0)	551 (53.2)	614 (58.7)
up to ¼ of the time	593 (28.5)	314 (30.3)	279 (26.7)
up to ½ of the time	171 (8.2)	94 (9.1)	77 (7.4)
up to ¾ of the time	64 (3.1)	27 (2.6)	37 (3.5)
more than ¾ the time, almost always	89 (4.3)	50 (4.8)	39 (3.7)
**working hours per week (** ${\rm{\bar x}}$ **, SD)**	37.4 (12.6)	43.1 (9.0)	31.8 (13.2)
**Musculoskeletal symptoms at follow-up, in 2017**			
**Neck**			
Yes	1137 (54.6)	463 (44.7)	674 (64.4)
No	945 (45.4)	573 (55.3)	372 (35.6)
**Shoulders**			
Yes	1102 (52.9)	498 (48.1)	604 (57.7)
No	980 (47.1)	538 (51.9)	442 (42.3)
**Upper back**			
Yes	635 (30.5)	261 (25.2)	374 (35.8)
No	1447 (69.5)	775 (74.8)	672 (64.2)
**Lower back**			
Yes	1177 (56.5)	581 (56.1)	596 (57.0)
No	905 (43.5)	455 (43.9)	450 (43.0)

n: number; ${\rm{\bar x}}$: mean; SD: standard deviation

### Associations between sitting at work and MSCs

Table 2 shows the association of all four regions of the musculoskeletal system at follow-up and baseline sitting at work. Increased weekly sitting times at work at baseline were rarely statistically significantly associated with MSCs at follow-up among the total population. PRs for the 12-month prevalence of neck, shoulder and lower back complaints were lower in highest sitting categories (model 3) compared with the moderate sitting categories, which may either suggest an inverse U-shaped relationship between sitting at work and neck and lower back pain or is an indication of selection bias (e.g., healthy worker effect).

**Table 2:** Associations between baseline workplace sitting in 2012 and complaints of neck, shoulder, upper back, and lower back pain in the last 12 months in 2017 (yes/no) among 2,082 employees aged 31–60 years in Germany. Poisson regressions. Prevalence Ratios (PR).

Exposure	12-month prevalence of complaints in different regions of the musculoskeletal system at follow-up							
Occupational sitting 2012	Neck *n* = 1137		Shoulder *n* = 1102		Upper back *n* = 635		Lower back *n* = 1177	
	PR	95% CI	PR	95% CI	PR	95% CI	PR	95% CI
**Model 1**								
Under 5 h/week	1	(Ref)	1	(Ref)	1	(Ref)	1	(Ref)
5 to < 15 h/week	0.99	0.88–1.11	0.98	0.88–1.10	0.99	0.82–1.18	0.95	0.85–1.05
15 to < 25 h/week	1.08	0.96–1.20	0.95	0.84–1.07	1.02	0.85–1.22	0.98	0.88–1.10
25 to < 35 h/week	1.11	0.99–1.24	0.92	0.81–1.04	0.90	0.74–1.11	0.91	0.81–1.02
35 or more hours/week	0.95	0.84–1.08	0.87	0.76–0.99	0.73	0.59–0.91	0.83	0.74–0.94
**Model 2**								
Under 5 h/week	1	(Ref)	1	(Ref)	1	(Ref)	1	(Ref)
5 to < 15 h/week	0.98	0.87–1.10	0.98	0.87–1.10	1.02	0.84–1.22	0.98	0.88–1.09
15 to < 25 h/week	1.07	0.95–1.20	0.95	0.84–1.07	1.07	0.88–1.29	1.04	0.93–1.17
25 to < 35 h/week	1.10	0.97–1.24	0.92	0.81–1.06	0.96	0.77–1.19	0.97	0.86–1.11
35 or more hours/week	0.95	0.83–1.09	0.88	0.76–1.01	0.78	0.62–0.99	0.90	0.79–1.03
**Model 3**								
Under 5 h/week	1	(Ref)	1	(Ref)	1	(Ref)	1	(Ref)
5 to < 15 h/week	1.01	0.90–1.13	1.03	0.92–1.15	1.10	0.92–1.32	1.02	0.92–1.14
15 to < 25 h/week	1.11	0.98–1.25	1.03	0.91–1.17	1.21	0.99–1.48	1.13	1.01–1.27
25 to < 35 h/week	1.14	1.00-1.31	1.02	0.88–1.17	1.11	0.88–1.39	1.09	0.95–1.24
35 or more hours/week	0.99	0.86–1.15	0.97	0.84–1.13	0.92	0.72–1.18	1.01	0.88–1.16

Model 1: unadjusted

Model 2: adjusted for sex, age, and occupational level

Model 3: adjusted for the covariates in model 2 and BMI, smoking, musculoskeletal diseases at baseline, psychological disorders, lifting/carrying heavy loads at work and leisure time physical activity

PR: prevalence ratio, Ref: reference category

### Regression analyses stratified by sex

Interaction tests revealed that sex did not significantly modify the associations between occupational sitting and MSCs (*p* > 0.05). However, stratifying by categories of sex showed mixed findings. The fully adjusted regression analyses of occupational sitting times at baseline and MSCs at follow-up (model 3) stratified by sex are shown in Tables 3 and 4, using “under 5 hours/week” of sitting at work as the reference category.

The results of the stratified analyses by sex demonstrated no discernible differences in the direction of the associations between occupational sitting at baseline and MSCs among men and women at follow up (Tables 3 and 4). We found indications of a difference between male and female workers for neck complaints at follow-up and between 25 and < 35 h/week of sitting at work, with men showing an increased prevalence of neck complaints that we did not observe for women. For shoulder complaints, no clear associations or patterns were observed for men or women (Table 3). However, there are differences in the peak PRs for the 12-month prevalence of upper back complaints between both women and men (Table 4). It is noteworthy that statistically significant PRs were not identified in any of the groups.

**Table 3:** Associations between baseline workplace sitting in 2012 and complaints of neck and shoulder pain in the last 12 months in 2017 (yes/no) stratified by sex to baseline among 2,082 employees aged 31–60 years in Germany. Poisson regressions. Prevalence Ration (PR).

Exposure	Strata defined by sex prior to baseline							
Occupational sitting 2012	Neck, *n* = 1137				Shoulder, *n* = 1102			
	Men		Women		Men		Women	
	PR	95% CI	PR	95% CI	PR	95% CI	PR	95% CI
**Model 3**								
Under 5 h/week	1	(Ref)	1	(Ref)	1	(Ref)	1	(Ref)
5 to < 15 h/week	0.84	0.67–1.06	1.12	0.98–1.27	0.99	0.83–1.19	1.05	0.90–1.21
15 to < 25 h/week	1.12	0.89–1.42	1.10	0.96–1.26	0.99	0.79–1.23	1.05	0.89–1.22
25 to < 35 h/week	1.26	1.00-1.60	1.06	0.91–1.24	1.00	0.80–1.26	1.01	0.84–1.22
35 or more hours/week	1.02	0.79–1.30	0.97	0.81–1.16	0.93	0.74–1.17	1.01	0.83–1.23

Model 3: adjusted for age, occupational level, BMI, smoking, musculoskeletal diseases at baseline, psychological disorders, lifting/carrying heavy loads at work and leisure time physical activity

PR: prevalence ratio, Ref: reference category

**Table 4:** Associations between baseline workplace sitting in 2012 and complaint of upper back and the lower back in the last 12 months in 2017 (yes/no) stratified by sex to baseline among 2,082 employees aged 31–60 years in Germany. Poisson regressions. Prevalence Ratios (PR).

Exposure	Strata defined by sex prior to baseline							
Occupational sitting 2012	Upper back, *n* = 635				Lower back, *n* = 1177			
	Men		Women		Men		Women	
	PR	95% CI	PR	95% CI	PR	95% CI	PR	95% CI
**Model 3**								
Under 5 h/week	1	(Ref)	1	(Ref)	1	(Ref)	1	(Ref)
5 to < 15 h/week	1.20	0.88–1.63	1.07	0.85–1.35	1.04	0.89–1.21	1.01	0.87–1.18
15 to < 25 h/week	1.15	0.79–1.66	1.25	0.99–1.59	1.13	0.95–1.34	1.13	0.97–1.32
25 to < 35 h/week	1.20	0.81–1.77	1.05	0.79–1.40	1.03	0.84–1.26	1.15	0.96–1.38
35 or more hours/week	1.08	0.74–1.59	0.75	0.52–1.07	1.00	0.83–1.21	1.03	0.84–1.27

Model 3: adjusted for age, occupational level, BMI, smoking, musculoskeletal diseases at baseline, psychological disorders, lifting/carrying heavy loads at work and leisure time physical activity

PR: prevalence ratio, Ref: reference category

### Leisure time physical activity stratification

Interaction tests revealed that leisure time physical activity did not significantly modify the associations between occupational sitting and MSCs (*p* > 0.05). However, stratifying by categories of physical activity showed mixed findings. The results from the regression analyses stratified by leisure time physical activity (models 1–3) are shown in Tables 5, 6, 7 and 8. For complaints of pain in the neck and shoulders, different frequencies of leisure time physical activity influenced the effect of sitting at work (Tables 5, 6, 7 and 8).

**Table 5:** Associations between baseline workplace sitting in 2012 and neck complaints in the last 12 months in 2017 (yes/no) stratified by leisure time physical activity to baseline among 2,082 employees aged 31–60 years in Germany. Poisson regressions. Prevalence Ratios (PR).

Exposure	Strata defined by leisure time physical activity prior to baseline					
	Neck, *n* = 1137					
Occupational sitting 2012	none of the time		little/some of the time		mostly/all the time	
	PR	95% CI	PR	95% CI	PR	95% CI
**Model 3**						
Under 5 h/week	1	(Ref)	1	(Ref)	1	(Ref)
5 to < 15 h/week	1.23	1.03–1.47	0.93	0.79–1.10	0.68	0.44–1.03
15 to < 25 h/week	1.17	0.96–1.43	1.10	0.93–1.30	0.97	0.65–1.45
25 to < 35 h/week	1.46	1.18–1.80	1.05	0.88–1.26	0.83	0.49–1.41
35 or more hours/week	1.20	0.96–1.49	0.90	0.74–1.10	0.66	0.30–1.43

Model 3: adjusted for sex, age, occupational level, BMI, smoking, musculoskeletal diseases at baseline, psychological disorders and lifting/carrying heavy loads at work

PR: prevalence ratio, Ref: reference category

**Table 6:** Associations between baseline workplace sitting in 2012 and shoulder complaints in the last 12 months in 2017 (yes/no) stratified by leisure time physical activity to baseline among 2,082 employees aged 31–60 years in Germany. Poisson regressions. Prevalence Ratios (PR)

Exposure	Strata defined by leisure time physical activity prior to baseline					
	Shoulder, *n* = 1102					
Occupational sitting 2012	none of the time		little/some of the time		mostly/all the time	
	PR	95% CI	PR	95% CI	PR	95% CI
**Model 3**						
Under 5 h/week	1	(Ref)	1	(Ref)	1	(Ref)
5 to < 15 h/week	1.12	0.94–1.34	1.02	0.87–1.21	0.75	0.54–1.05
15 to < 25 h/week	1.12	0.91–1.38	1.05	0.88–1.26	0.69	0.46–1.02
25 to < 35 h/week	1.30	1.03–1.64	0.95	0.77–1.16	0.73	0.45–1.19
35 or more hours/week	1.12	0.90–1.41	0.94	0.77–1.16	0.58	0.27–1.22

Model 3: adjusted for the sex, age, occupational level, BMI, smoking, musculoskeletal diseases at baseline, psychological disorders and lifting/carrying heavy loads at work

PR: prevalence ratio, Ref: reference category

**Table 7:** Associations between baseline workplace sitting in 2012 and upper back complaints in the last 12 months in 2017 (yes/no) stratified by leisure time physical activity to baseline among 2,082 employees aged 31–60 years in Germany. Poisson regressions. Prevalence Rations (PR).

Exposure	Strata defined by leisure time physical activity prior to baseline					
	Upper back, *n* = 635					
Occupational sitting 2012	none of the time		little/some of the time		mostly/all the time	
	PR	95% CI	PR	95% CI	PR	95% CI
**Model 3**						
Under 5 h/week	1	(Ref)	1	(Ref)	1	(Ref)
5 to < 15 h/week	1.24	0.92–1.66	1.11	0.85–1.44	0.70	0.37–1.32
15 to < 25 h/week	1.25	0.88–1.76	1.28	0.97–1.69	1.07	0.61–1.90
25 to < 35 h/week	1.20	0.81–1.78	1.14	0.84–1.56	1.03	0.46–2.32
35 or more hours/week	1.04	0.72–1.52	0.93	0.66–1.30	0.28	0.04–1.96

Model 3: adjusted for sex, age, occupational level BMI, smoking, musculoskeletal diseases at baseline, psychological disorders and lifting/carrying heavy loads at work

PR: prevalence ratio, Ref.: reference category

An association was observed between occupational sitting at baseline and the 12-month prevalence of neck, shoulder and lower back complaints among workers who did not engage in leisure time physical activity. Specifically, statistically significant increased PRs were observed for neck and shoulder complaints in association with 25 to < 35 h/week of occupational sitting among people reporting no strenuous leisure time physical activities. For lower back complaints, increased PRs were observed for both 15 to < 25 and 25 to < 35 h/week of occupational sitting (Table 8). However, other stratifications with little or some time, and mostly or all the time showed no increased risk.

Among workers with no leisure time physical activity, the highest PR was estimated for neck complaints for those sitting 25 to < 35 h/week at work after adjusting for all covariates (PR 1.46; 95% CI 1.18–1.80) (Table 5). Similar associations were not observed among groups of workers engaging in at least a little physical activity in their free time (little or some time, and mostly or all the time). Some indications of effect modifications were observed between physical activity category and occupational sitting for upper back complaints but these were not consistent and did not reach statistical significance (Table 7).

**Table 8:** Associations between baseline workplace sitting in 2012 and lower back complaints in the last 12 months in 2017 (yes/no) stratified by leisure time physical activity to baseline among 2,082 employees aged 31–60 years in Germany. Poisson regressions. Prevalence Rations (PR)

Exposure	Strata defined by leisure time physical activity prior to baseline					
	Lower back, *n* = 1177					
Occupational sitting 2012	none of the time		little/some of the time		mostly/all the time	
	PR	95% CI	PR	95% CI	PR	95% CI
**Model 3**						
Under 5 h/week	1	(Ref)	1	(Ref)	1	(Ref)
5 to < 15 h/week	1.15	0.98–1.36	1.00	0.86–1.17	0.71	0.50–1.01
15 to < 25 h/week	1.22	1.01–1.47	1.13	0.96–1.33	0.80	0.57–1.12
25 to < 35 h/week	1.32	1.07–1.63	1.03	0.85–1.24	0.88	0.54–1.41
35 or more hours/week	1.08	0.87–1.43	1.03	0.85–1.25	0.66	0.34–1.29

Model 3: adjusted for sex, age, occupational level, BMI, smoking, musculoskeletal diseases at baseline, psychological disorders and lifting/carrying heavy loads at work

PR: prevalence ratio, Ref.: reference category

## Discussion

### Main results

The aim of this study was to examine the relationship between occupational sitting and the 12-month prevalence of neck, shoulder, upper back, and low back complaints among German workers. To the best of our knowledge, this is the only prospective study examining the association between sedentary working times and MSCs in a representative population of German workers. More than 60% of the study population self-reported spending over half of their working hours in a sitting position. Using an estimation of the weekly working hours spent sitting, our findings indicated that sitting at baseline was associated with an increased 12-month prevalence of neck, shoulder and low back complaints at follow-up if workers did not report any strenuous leisure time physical activities at baseline. Otherwise, we did not observe clear dose-response patterns between sitting time and MSC, with PRs sometimes even sinking below 1 for the highest exposure category. When analyzing the data separately for men and women, no clear differences between men and women were observed, but men with an estimated weekly occupational sitting time of 25 to < 35 h/week had an increased prevalence of neck complaints (PR 1.26, 95% CI 1.00-1.60) that we did not observe for women (PR 1.06, 95% CI 0.91–1.24). However, this could be a chance finding.

### Comparison to other studies

From our findings, we cannot conclusively determine whether sitting per se is a direct risk factor for MSCs, or if sitting (as it was asked here) serves as a proxy for other unaddressed risk factors, such as seated restricted/awkward body postures (e.g., bent forward over work or microscope). Systematic reviews that focused on longitudinal studies investigating occupational risk factors for neck-shoulder complaints found strong evidence supporting a causal relationship between awkward body postures and neck-shoulder pain [10, 62]. However, the evidence regarding sitting as a risk factor was considered insufficient in those reviews. Some longitudinal studies show a negative association between sitting time and MSCs [63, 64]. However, prospective studies specifically examining occupational sitting and complaints related to the neck, shoulder, upper back, and lower back are limited and have yielded conflicting results [65, 66], leaving the role of sitting time in MSCs not entirely clear.

Our findings regarding the association between occupational sitting time and neck/shoulder complaints, differ some from the results of the studies by Grooten et al. [63] and Picavet et al. [64]. Grooten et al. [63] assessed the sitting duration of 803 workers through self-reporting and found that sitting for more than 75% of working hours was associated with a higher likelihood of being free of neck/shoulder pain after 5 years. We observed increased PRs for neck complaints at the 5-year follow-up that approached but did not reach statistically significance. Similarly, Picavet et al. [64] reported that increased self-reported time spent sitting at work was associated with reduced upper extremity pain over a 15-year period. However, Hallman et al. found an inverse U-shaped relationship but no significant association between objectively measured sitting time at work and neck/shoulder complaints among blue-collar workers in their study [18]. They suggested that this finding might be attributed to a healthy worker effect, indicating that workers experiencing severe pain may have shifted to work tasks involving less prolonged sitting. On the other hand, our findings are in line with the study conducted by Ariëns et al. [66] using video observations to estimate PRs for sitting time and neck pain for a sample of 977 workers from various occupations. They discovered that sitting for more than 95% of working hours increased the risk of reporting regular or persistent neck pain three years later, compared to those who engaged in very little sitting.

Cervical disc disorders (ICD-10: M50), a relatively clearly definable disease presenting with neck pain may also be associated with occupational sitting. In fact, a case-control study of 226 participants conducted by Elsner and colleagues found an increased odds of cervical disc prolapse associated with computer work for categories of cumulative hours of computer work up to 4,945 h and between 4,945 and < 13,883 h versus no computer work [67]. Further research of long-term cumulative exposures to computer work and the ergonomic characteristics of computer work are needed to confirm these results.

As for upper back complaints, we observed some increased PRs for categories of sitting times, albeit not statistically significant and without any clear trends. Our observations differ from those of Hossain et al. [68], who examined the prevalence of work-related musculoskeletal disorders among readymade garment workers in Bangladesh, and found a significant association between work-related sitting and upper back disorders. This difference may be due to the use of different types of instruments to measure risk factors between studies. In particular, Hossain et al. used the Quick Exposure Check instrument to assess work-related ergonomic risk factors and symptoms by body region, such as upper back pain.

In terms of low back complaints, a cross-sectional analysis in the ‘New method for Objective Measurements of physical Activity in Daily living (NOMAD)’ in Denmark study revealed a significant positive association between total sitting time (per hour) and the intensity of low back pain (odds ratio; OR = 1.43, 95%CI = 1.15–1.77) [69]. Our study also found a statistically significant increased PR for low back complaints, but only in the category of 15 to < 25 h/week of occupational sitting. However, we did considered only the prevalence of complaints and not the intensity of pain.

Although we did not detect any discernible disparities between men and women in our findings, it is important to underscore a discovery made in previous studies. A cross-sectional study in four age groups by Spittaels et al. [70] unveiled sex-dependent variations in sedentary time and levels of physical activity across different age groups. Specifically, their results depicted a linear relationship among men, where sedentary behavior increased and physical activity decreased with age, culminating in a peak of 59% time spent sedentary in adults. This avenue of investigation may shed further light on the reasons behind the varying effects of prolonged sitting across different contexts, such as work and leisure, and among distinct sexes. For instance, Toomingas et al. [71] observed that women spent an average of 11% of their workday engaged in uninterrupted sitting for more than an hour, whereas men spent only 4.6% of their workday in such long sitting bouts (*p* = 0.013).

When stratifying for leisure time physical activity, we observed differences in the association between sitting at work and the 12-month prevalence of shoulder and neck pain among workers who do not engage in leisure time physical activity in their free time and those who are physically active during their leisure time. This is similar to findings of a study examining full-time workers in Brisbane, Australia and Daegu, South Korea, which found that increased physical activity and less time sitting had a beneficial effect on reducing the development of neck pain [72]. A longitudinal study of asymptomatic office workers also found that for every 1,000 steps of walking, the risk of neck pain reduced by 14% [73]. However, these differences were not evident for lower back complaints. These results support previous findings from the systematic reviews conducted by Kwon et al. [74] and Swain et al. [75].

Occupational and leisure physical activity/sedentary time have divergent impacts on health. A survey of Danish workers found sedentary workers were more likely to have low back pain if they did not participate in sports, and workers with high physical workloads had more complaints in the lower-extremities if they did not participate in sports [76]. Holtermann et al. [77] also describe a paradox where the risk of long-term sickness absences increases with occupational physical activity and decreases with leisure time physical activity.

### Strengths and weaknesses

The strengths of this study include the S-MGA’s the utilization of the Federal Employment Agency’s register as a comprehensive sampling framework. The use of the register provides a clear and unambiguous definition of the population under study, and the population-based registry contains the complete records of all workers included in the Germany social security system [35]. This helped ensure a representative study population.

One of the other strengths of this study was the adjustment of individual and work-related risk factors as potential confounders of MSCs. We also adjusted for lifting/carrying of heavy objects as a risk factor for MSCs. Nevertheless, it is still plausible that other unadjusted factors at work or during leisure may have influenced the association between sitting and MSCs, such as socioeconomic status and awkward body postures [45, 65, 78].

Although we had longitudinal data, we could not assess incident MSCs, because prevalent MSC at baseline could not be excluded from the analysis. However, MSCs vary over time, making it difficult to consider their incidence. Nevertheless, we attempted to mitigate any potential reverse causality by using baseline exposure and covariate data, and by adjusting for pre-existing musculoskeletal conditions.

In addition, while information on awkward body postures were available, we chose not to adjust for it because the question did not adequately differentiate between seated restricted/awkward positions (bent forward) and other awkward positions, such as reaching behind the head and twisting at the waist. Furthermore, based on the results from a systematic review [25], there was no evidence of an association between awkward occupational postures and LBP. It is possible that awkward occupational postures while sitting are independently causative of LBP in the populations of workers studied [25]. Nevertheless, the addition of neck and arm posture measurements could provide valuable insights into whether sitting posture primarily represents postural restriction or if it genuinely acts as a risk factor for MSCs [18]. Future studies should simultaneously record both sitting and arm postures and incorporate both variables into a model aiming to explain the risk of MSCs. These models could also explore the possibility of an interaction between sitting posture and arm postures.

Selection bias may also be a limitation of our analysis. For one, the only suitable sampling frame available for finding a rather representative German working population is the social security system. However, civil servants, the self-employed and freelancers are exempt from the social security system in Germany. Thus, these workers were not included in the S-MGA. Also, the S-MGA did not comprise workers older than 60 years at baseline because persons over 60 would reach retirement age of 65 before the first follow-up. We also excluded those who were retired or were otherwise not employed at follow-up, which may have introduced healthy work bias, as health problems can lead to unemployment or early retirement [79]. Although this may introduce some healthy-worker bias, we focused on those who were still working because sitting at work may have more of an acute or more immediate effect on the prevalence of MSCs. We found a strong correlation between baseline and follow-up values of sitting at work (Spearman’s rho = 0.8, *P* < 0.001) and a moderate correlation for working hours (Spearman’s rho = 0.7, *P* < 0.001).

One potential reason for the inconsistent findings among studies could be the use of self-reported measurements of sitting duration, which may be subject to inaccuracies and biases, possibly influenced by factors such as musculoskeletal complaints [69, 80]. Although sitting time is a prevalent behavior that can be easily recalled in interviews [81], it has been suggested that self-reported measures of sedentary behavior may be less reliable and valid than objective measures [82]. Objective data on sitting would have also allowed for a consideration of sitting in relation to standing or movements at work.

Furthermore, self-reported sitting time is prone to underestimation [83]. A further limitation is the self-reported information on physical activity. Self-reported assessments have been shown to correlate poorly with objective measures, and discrepancies seem to increase with the intensity of the physical activity being assessed [84]. This may have influenced our results as we used a variable describing activities with increased physical exertion. While measurement errors are likely, they would more likely be non-differential, which may bias the risk estimate towards null effect. If there is non-differential exposure misclassification, the true association between sitting time and MSCs might be even stronger than shown in our study.

Another limitation was that the S-MGA faced challenges in achieving the targeted response rate of 50%, despite considerable efforts made (e.g., reminder letters, incentives). The actual response at baseline and follow-up was about 37.5% and 59%, respectively. This low response might have implications for the internal as well as external validity of the study, as respondents might be less representative of the general population compared to the baseline cohort. Attrition over time further exacerbates this issue, potentially leading to selection effects.

### Implications

Our study suggests that even moderate physical activity can help alleviate the negative impact of prolonged sitting on the risk of MSCs. This implies that promoting physical activity or integrating movement-based tasks into work activities could serve as effective methods for primary prevention of MSCs. In a recent systematic review conducted by Sundstrup et al. on workplace interventions to address musculoskeletal disorders among employees engaged in physically demanding work, they underscored the significance of implementing physical exercise at the workplace to reduce MSCs, particularly if it aligns with the job context [85]. Additionally, in a review focusing on workplace interventions for preventing upper extremity musculoskeletal disorders and symptoms, Eerd and colleagues found that stretching exercise programs, vibration feedback on mouse use, and workstation forearm supports demonstrated a moderate level of evidence for positively affecting the prevention of upper extremity MSCs. They further recommend the incorporation of workplace-based resistance training exercise programs to prevent and manage symptoms and disorders related to upper extremity MSCs [86].

One aspect that requires further investigation is how changes to working conditions following the pandemic may have changed the relationship between sedentary work and MSCs. This should be possible when the next wave of S-MGA data is available.

Moreover, studies by Gupta et al. [69] and Nourbakhsh et al. [87] have indicated that occupational sitting, leisure time sitting, and total sitting may be correlated. Hence, it may be important either to perform a combined analysis of occupational and leisure time sitting or to mutually adjust for these variables when investigating their independent association with MSCs. Another reason for differentiating between work and leisure time sitting is the potential variation in temporal patterns of sitting, which can be relevant for both metabolic [88] and musculoskeletal symptoms [89]. Additionally, MSCs often coexist with multiple health issues and morbidities [8, 90] and are becoming increasingly common comorbidities in chronic diseases like type 2 diabetes [91, 92].

## Conclusion

These findings suggest a complex and multifactorial relationship between occupational sitting and MSCs, potentially involving interactions with other leisure time physical activity. While providing valuable insights, our study emphasizes the necessity for further research to fully elucidate mechanisms and establish a definitive causal relationship. It is crucial to either conduct a combined analysis of occupational and leisure time sitting or mutually adjust for these variables to explore their independent association with MSCs.

Furthermore, our results suggest that even infrequent physical activity may be an effective intervention or prevention strategy against sitting-related MSC in workers. However, further research is needed on the intensity, frequency and duration that may be beneficial for prevention. The study illuminates intricate associations between workplace sitting and various types of pain, hinting at potential protective effects within specific subgroups. These findings underscore the need for comprehensive research to understand these relationships better and identify preventive measures for workplace-related pain complaints. Future studies should delve into additional factors and utilize more objective measures of sitting and leisure time physical activity to enhance result validity and uncover underlying mechanisms. Addressing these knowledge gaps will enable the development of targeted interventions promoting musculoskeletal health and overall well-being in work environments.

### Electronic supplementary material

Below is the link to the electronic supplementary material.

Supplementary Material 1

## Data Availability

A file called the Scientific Use File (SUF), which includes both wave 1 and wave 2 of the cohort, is accessible at the Research Data Centre of the Federal Institute of Occupational Safety and Health (FDZ-BAuA). Research teams interested in analyzing data from the S-MGA study can request access through the FDZ-BAuA (https://www.baua.de/DE/Forschung/Forschungsdaten/Forschungsdaten_node.html).
