# Supplementary figures and images for: Impact of sitting at work on musculoskeletal complaints of German workers - results from the study on mental health at work (S-MGA)
Source: J Occup Med Toxicol. 2024 Mar 27;19:9. doi: 10.1186/s12995-024-00408-7 (PMC10967152; doi:10.1186/s12995-024-00408-7)

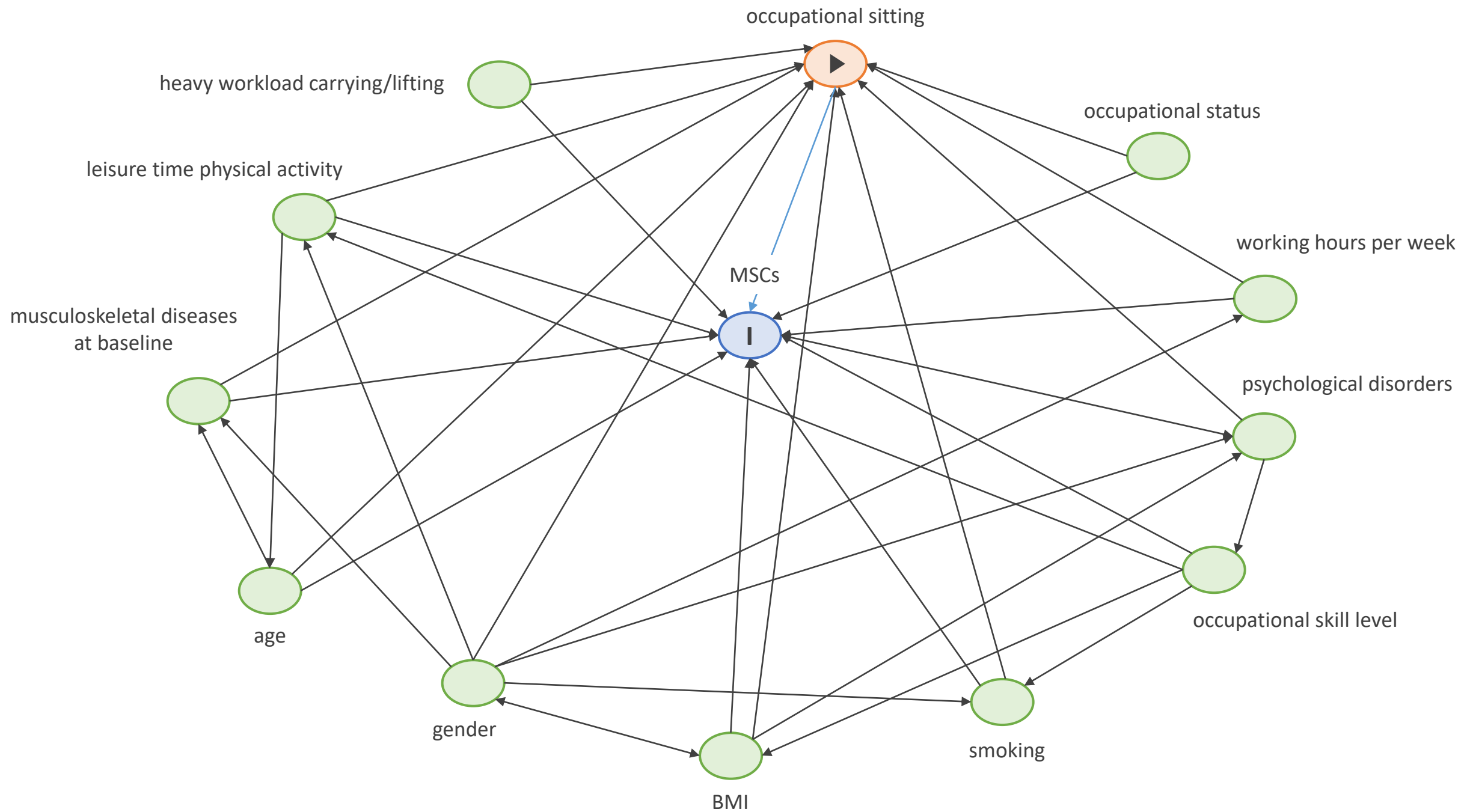

Supplement: Supplementary file 1 — Supplementary Material 1 [file 12995_2024_408_MOESM1_ESM.pdf]
